# Cognitive Control, Cognitive Biases and Emotion Regulation in Depression: A New Proposal for an Integrative Interplay Model

**DOI:** 10.3389/fpsyg.2021.628416

**Published:** 2021-04-30

**Authors:** Dolores Villalobos, Javier Pacios, Carmelo Vázquez

**Affiliations:** ^1^Department of Experimental Psychology, School of Psychology, Complutense University of Madrid, Madrid, Spain; ^2^Laboratory of Cognitive and Computational Neuroscience, Center for Biomedical Technology, Technical University of Madrid, Madrid, Spain; ^3^Department of Clinical Psychology, School of Psychology, Complutense University of Madrid, Madrid, Spain

**Keywords:** depression, cognitive control, shifting, updating, inhibition, cognitive biases, rumination, emotion regulation

## Abstract

Research traditions on cognition and depression focus on relatively unconnected aspects of cognitive functioning. On one hand, the neuropsychological perspective has concentrated on cognitive control difficulties as a prominent feature of this condition. On the other hand, the clinical psychology perspective has focused on cognitive biases and repetitive negative patterns of thinking (i.e., rumination) for emotional information. A review of the literature from both fields reveals that difficulties are more evident for mood-congruent materials, suggesting that cognitive control difficulties interact with cognitive biases to hinder cognitive switching, working memory updating, and inhibition of irrelevant information. Connecting research from these two traditions, we propose a novel integrative cognitive model of depression in which the interplay between mood-congruent cognitive control difficulties, cognitive biases, and rumination may ultimately lead to ineffective emotion-regulation strategies to downregulate negative mood and upregulate positive mood.

## Introduction

The World Health Organization identifies depression as one of the most prevalent disorders worldwide, affecting more than 300 million people. Depression is the third cause of disability in the world, and it is expected that it will become the first cause of disability in high-income countries by 2030 (Mathers and Loncar, 2006). In addition, the nature of depression makes it a highly recurrent condition with a probability of recurrence between 40 and 60% after a first episode, a probability that rises to 90% from the third episode onward (Bockting et al., 2015).

Therefore, depression is considered a disorder that requires a global approach from different perspectives, paying special attention to the development of preventive strategies (Muñoz and Bunge, 2016). A better understanding of the causes of depression and the mechanisms involved in its onset and maintenance is essential to move in this direction.

According to the Diagnostic and Statistical Manual of Mental Disorders (American Psychiatric Association DSM 5 Task Force, 2013), major depressive disorder (MDD) is characterized by different signs and symptoms, most of them related to the individual's emotional state (e.g., pessimism, feelings of worthlessness or excessive guilt, marked loss of interest, recurrent thoughts of death) with the necessary presence of either a depressed mood and/or a decrease ability to experience pleasure. Therefore, depression is characterized by a negative mood or a diminished positive mood. As it is considered in the current review, this dual mood perspective on depression has important consequences to guide and interpret findings from research on this disorder (Hayes et al., 2015). Based on the centrality of emotional components in depression, there has been abundant research addressing the etiological analysis of depression from the perspective of emotion dysregulation (Joormann and Stanton, 2016; Visted et al., 2018). In fact, numerous researchers suggest that people who are not able to regulate and diminish negative affective states and to maintain or upregulate positive affective states are the most vulnerable to initiate or maintain depressive episodes (Liu and Thompson, 2017).

### Emotion Regulation, Cognitive Control, and Cognitive Biases

Emotion regulation is a key concept to consider when addressing depression. Emotion regulation occurs when one activates a goal to influence the emotion generative process (Gross and Jazaieri, 2014); in other words, “it is an activation of a goal that recruits one or more processes to influence emotion generation” (Gross et al., 2011). These processes, which can be either deliberate and effortful (explicit) or unconscious and effortless (implicit), have the aim to decrease (downregulate) or increase (upregulate) different facets of emotional responding (e.g., intensity, duration, or frequency) (Sheppes et al., 2015). Thus, a key theoretical piece of emotion-regulation theories is that people do not passively experience their emotions. Instead, people actively respond to their emotional state and, in some circumstances, try to modify it (Joormann and Stanton, 2016). Hence, it is key to consider personal skills to generate such emotion-regulation responses as depressed individuals often show difficulties adequately regulating their mood (Joormann and Stanton, 2016).

Research carried out in recent decades focuses on analyzing cognitive functioning in people with depression, and it is now widely accepted that MDD is characterized by cognitive impairment, especially in acute phases (Hammar and Ardal, 2009). There is also cross-national evidence showing that problems in cognition (specifically, difficulties in attention and learning of new tasks) are considered by patients with depression as one important burdensome problem (at the same level as sleep problems and lack of energy although perceived as less problematic than problems in affect, domestic life, work, and interpersonal activities) (Kamenov et al., 2016). In fact, the DSM-5 includes “a slowing down of thought” or “reduced ability to think or concentrate” among the cognitive symptoms that are required for the diagnosis of MDD, together with the emotional state symptoms previously described (American Psychiatric Association DSM 5 Task Force, 2013). Unfortunately, despite more than 90% of patients with a history of depression reporting significant cognitive difficulties in their daily living activities, only 50% say that these impairments have been explored by mental health professionals (Clark Health Communication, 2015). In light of this situation, it is recommended that interventions to remediate cognitive functioning in depression should be considered a priority (National Academies of Sciences Engineering Medicine, 2015).

Depressed individuals may show deficits in different domains of cognitive functioning, particularly in executive function, attention, memory, and psychomotor speed (Marazziti et al., 2010). These deficits are often found in many studies despite differences in tools or procedures to assess cognitive functioning, including classical neuropsychological tests (Hammar and Ardal, 2009) and computerized batteries, such as the Cambridge Neuropsychological Test Automated Battery (CANTAB) (Rock et al., 2014). These “cold” cognition (Roiser and Sahakian, 2013) deficits also appear in patients experiencing a first depressive episode (Lee et al., 2012) and are one of the most frequent complaints of patients in clinical settings (Gonda et al., 2015). Most importantly, these deficits particularly emerge in effortful processes rather than in automatic ones (Hartlage et al., 1993). Effortful processing requires voluntary deployment of attention and thereby takes place serially, inhibits other pathways, and is influenced by cognitive capacity limitations. Therefore, depressed people show deficits in planning, initializing, and monitoring complex goal-directed behaviors, especially when coping with emotionally negative distractors (Vazquez and Hernangómez, 2009). These difficulties often pose exhausting demands to depressed individuals if they wish to overcome or compensate those deficits by, for instance, making additional efforts to maintain their attention (Beevers, 2005). Hence, cognitive control, an essential component of executive function, seems to be compromised in people suffering from depression.

Together with cognitive control difficulties, early cognitive models of depression emphasize the causal role of cognitive biases in this disorder (Beck, 1967). Since then, much research consistently shows that a distorted elaboration of information leads depressed individuals to view themselves and their environment in a negative way, contributing to the development, maintenance, and recurrence of depression (Kircanski et al., 2012). There is robust meta-analytic evidence on the negative cognitive biases in experimental tasks of attention (Peckham et al., 2010; Armstrong and Olatunji, 2012; Epp et al., 2012), memory (Matt et al., 1992), and interpretation (Everaert et al., 2017a) as well as in self-report questionnaires that assess biases, such as dichotomous thinking, catastrophizing, overgeneralizing, or magnification of the negative and minimization of the positive, in depressed individuals (Nieto et al., 2020). Nevertheless, the relationship among these biases is not yet well-understood. For one thing, although cognitive biases in attention, interpretation, and memory processes are mostly investigated in isolation, recent studies suggest that complex interactions among them best account for depression severity and diagnostic status (i.e., “the combined cognitive biases hypothesis”; Everaert et al., 2014; Sanchez et al., 2017). Interestingly, a functional and structural neurobiological architecture is proposed to underlie the mutual interaction between bottom-up processing and top-down control that facilitates the onset and maintenance of cognitive biases. In their model, Disner et al. (2011) propose that the activation of negative self-referential schemas [presumably established early in life; see Beck (2008) for a developmental perspective] influences information processing by exacerbating bottom-up pathways involved in emotion processing. Complementarily, an attenuated function of top-down systems would prevent depressed individuals from restraining the heightened processing of emotional stimuli. Bottom-up dynamics include hyperactivity in the thalamus (Greicius et al., 2007) and enhanced and long-lasting amygdala reactivity (Drevets, 2001) as well as abnormalities in the subgenual cingulate cortex, where emotional feedback from the limbic system is relayed to higher order cortical regions (Greicius et al., 2007). Dysfunctional top-down control would consist of decreased functioning of ventrolateral prefrontal cortex (Beevers et al., 2010) and abnormalities in the dorsolateral prefrontal cortex (Fales et al., 2008; Li et al., 2010), including a decoupling between its left counterpart and the amygdala (Davidson, 2000; Drevets, 2001) and in the anterior cingulate cortex (Mitterschiffthaler et al., 2008; Eugène et al., 2010). In this view, reduced cognitive control would interact with cognitive biases to enhance the processing of negative information and would also be a mechanism itself involved in the maintenance of those cognitive biases. These complex interactions may ultimately promote ineffective use of emotion-regulation strategies (Joormann, 2010; Grahek et al., 2018), which, coupled with abnormal styles of processing information, such as having a repetitive or ruminative style of thinking, may contribute to maintaining negative affect and reducing positive affect (Joormann and Vanderlind, 2014). Also, whether these biases remain stable over time or are simply associated with other symptoms of depression is still relatively unknown, and there are inconclusive results. For instance, whereas some studies find that both currently and remitted depressed individuals have poorer performance in attentional and memory tasks (Gupta and Kar, 2008, 2012), other studies find that remitted depressed patients have similar scores on a scale of dysfunctional negative thoughts than depressed participants (Haeffel et al., 2005) or than healthy controls (Meites et al., 2008); also, other studies show that effective psychological therapies reduce negative attentional biases, measured with eye-tracking techniques, toward emotional information (Vazquez et al., 2018), which supports the idea that these cognitive elements are not stable markers of depression. Interestingly, these results support, in principle, the plausibility of using intervention methods directly aimed at modifying cognitive biases.

Although the present review is focused on cognitive control difficulties, cognitive biases, and a repetitive mode of processing, there are also some relevant potential cognitive elements that may play a role in depression. For instance, metacognitive models of depression and other emotional disorders emphasize that beliefs about thoughts and emotions may also contribute to increased emotional distress. Wells (2009, 2013), in his influential self-regulatory executive function (S-REF) model, proposes that negative thoughts and beliefs about oneself or the world may be amplified if the individual believes that holding those beliefs is useful (i.e., a metacognitive belief). This negative metacognition may diminish attentional control, thereby reducing the probability of disengaging from those repetitive negative thoughts and interpretations as well as hindering the ability to retrieve specific autobiographical memories. According to this model, interventions aimed at improving attentional control itself should reduce metacognitions, thereby reducing emotional symptoms.

### The Present Review

To further explore the potential interactions between cognitive control deficits and cognitive biases, we review evidence of cognitive control impairments in depression under conditions of processing neutral (i.e., cold cognition) or emotional information (i.e., hot cognition). Given the complexity of the cognitive control construct, we scrutinize its main constituents “as originally proposed by Miyake et al. (2000)” in research with depressed individuals. Moreover, we present current evidence on the linkage between the extent of cognitive control difficulties and the severity of depression. Finally, we review evidence that cognitive control difficulties in depression are ultimately related to the use of maladaptive emotion-regulation strategies. In particular, we focus on ruminative styles of responding because they are proposed to be the most frequent maladaptive mode of processing for emotion regulation (Visted et al., 2018). After reviewing and synthesizing the evidence gathered in these fields, which have been scarcely interconnected in the extant literature, we ultimately propose an integrative cognitive model of depression in which the interplay between cognitive control difficulties, cognitive biases, and rumination may be a key constituent of ineffective emotion-regulation strategies to downregulate negative mood and upregulate positive mood.

## Cognitive Control and Depression

Executive functions (Stordal et al., 2005; Snyder, 2013) are among the cognitive processes that have been commonly found to be affected in people with depression. Executive functions are high-level cognitive processes that, through their influence on lower-level processes, allow individuals to regulate their thoughts and actions in goal-directed behaviors (Friedman and Miyake, 2017). Executive functions encompass different skills or abilities, such as prioritization and sequencing of behaviors, inhibition of automatic or stereotyped behaviors, knowledge about what information is most relevant for the achievement of current goals (i.e., resisting potentially distracting information), alternation between tasks, use of relevant information for decision making, and categorization and management of novel information or situations (Banich, 2009).

Cognitive control is considered a key component of executive function. It is described as the ability to selectively attend to relevant stimuli and goals and inhibit the processing or responding to non-relevant stimuli (Wante et al., 2017). This ability, therefore, refers to the set of cognitive processes that allow a flexible adaptation of cognition and behavior according to the individual's main objectives (Friedman and Miyake, 2017). Cognitive control processes help to optimize behavior in situations in which action trends prompt conflict and, hence, must be modified according to contextual information (Paulus, 2015). Consequently, cognitive control comprises a series of processes that regulate the content of working memory (Everaert et al., 2017b). These processes are also related to the ability to flexibly engage in different thoughts and behaviors that fit situational demands, which research shows to be problematic in individuals with depression (Cheng et al., 2014; Stange et al., 2017).

Although several components and mechanisms of cognitive control are proposed in the literature, Miyake's three-component model of executive functioning (Miyake et al., 2000), later referred to as “the unity/diversity model” (Miyake et al., 2000; Miyake and Friedman, 2012), might be an adequate heuristic to articulate the extant knowledge on cognitive control. The first and more used model proposes three differentiated processes in cognitive control: (a) *shifting* (i.e., the ability to change or alternate between tasks or mental sets), (b) *updating* (i.e., the ability to refresh and monitor information representations in working memory), and (c) *inhibition* (i.e., the ability to suppress or override dominant responses). Yet these components are not completely separated one from another. Even at the definition level, these concepts overlap to some extent in terms of the mechanisms involved in each one (Koch et al., 2010; Yoon et al., 2014). Although Miyake's model is not exempt from limitations (Grahek et al., 2018), two decades after its publication, this model has proven itself useful in addressing alterations in cognitive control in different conditions: autism (Poljac and Bekkering, 2012), obsessive compulsive disorder (Kalanthroff et al., 2017), attention deficit and hyperactivity disorder (Bueno et al., 2017), anxiety (Liang, 2018), fibromyalgia (Munoz Ladron de Guevara et al., 2018), and depression (De Lissnyder et al., 2012b; Snyder, 2013; Joormann and Tanovic, 2015; Everaert et al., 2017b) and even in research focused on intervention programs for depression (Koster et al., 2017).

In the following sections, studies on cognitive control in depression, published since the model was formulated (Miyake et al., 2000), are reviewed. For practical reasons, we focus on the original model to group the diversity of studies on executive function in depression within the three main components first proposed. Additionally, with the aim of exploring the modulatory effect of emotions in cognitive control operations in depression and following the “cold *vs*. hot” cognition distinction (Roiser and Sahakian, 2013), the review examines studies conducted with non-emotional or emotional stimuli. This distinction is relevant as there is evidence showing that even performance in cold cognitive tasks may be affected by emotional cognitions aroused by performing the task itself (e.g., sense of failure, worthlessness, hopelessness, etc.) [see a review in Elliott et al. (2011), Robinson et al. (2016)]. Therefore, we conducted a literature search on PubMed and Scopus databases using the search terms “Depression” OR “Depressive” AND “Cognitive control” OR “Shifting” OR “Updating” OR “Inhibition.” We limited our search to the years 2000–2017 for peer-reviewed articles in English. Abstracts of retrieved articles were examined and included if they met all the following criteria: (a) adult patients diagnosed with MDD, (b) assessment of cognitive functioning through neuropsychological instruments or experimental tasks, and (c) focus mainly on behavioral measures. After removing duplicates, the literature search identified 293 publications. Based on the titles screening first, second on the abstract reading, and then applying the inclusion/exclusion criteria, the total number of remaining articles was 62. During this screening process, 13 additional articles were identified in the reference lists of those papers in the initial set and were, therefore, incorporated into the narrative.

### Shifting and Depression

Also known as switching (Ravizza and Carter, 2009), shifting can be defined as the ability to flexibly alternate between different tasks or mental sets (Miyake and Friedman, 2012). Set shifting encompasses different executive processes, such as alternating attention between different aspects of the stimuli, following changing instructions, recovering such instructions from memory, and behaving accordingly while inhibiting the instructions from the previous task and, simultaneously, performing a general supervision of the task (Grönholm-Nyman et al., 2017). Shifting can be regarded as an important contributor to adaptive emotion regulation skills in depression as it allows individuals to move their attention away from information that is not relevant at the moment (e.g., negative emotional material) and focus on positive or relevant materials to achieve effective emotion regulation (Joormann and Tanovic, 2015).

Although most neuropsychological tests are highly demanding for the individual and involve more than one specific cognitive function, it is usually accepted that shifting can be adequately measured by two classical tools (Arbuthnott and Frank, 2000; Wilmsmeier et al., 2010; Snyder, 2013): the Wisconsin Card Sorting Test (WCST) (Lezak, 2012) and the Trail Making Test part B (TMT-B) (Reitan, 1958). The assessment of patients with MDD employing these tests has evidenced shifting deficits (Harvey et al., 2004), independently of the subtype (melancholic, atypical, or undifferentiated) and phase of depression (during the acute depressive episode or in the remission phase) (Lin et al., 2014). Nevertheless, it is suggested that the effects observed in the TMT-B test in a population with MDD could be explained by a slowdown in processing speed, evident in part A (in which participants are required to connect numbered circles, 1–25, distributed over a sheet of paper), rather than by a specific deficit in shifting processes (as indexed in part B, in which participants are required to connect numbers and lettered circles alternating between them, e.g., 1-A-2-B-3-C, etc.) (Snyder, 2013).

Using experimental tasks, Murphy et al. (2012) compared the shifting skills of patients with MDD and healthy volunteers by using two versions of the Go/No-Go Affective Shifting Task: neutral and emotional. The classical (neutral) version of this task consists of pressing a key as quickly as possible when the target stimulus appears (e.g., a prespecified letter) and not pressing it when a distractor appears (e.g., a number). In the emotional version, the structure and instructions are the same with the exception that the stimuli (e.g., words) have a positive or negative emotional content. These authors found that, in the neutral version of the task, the ability of patients with MDD to flexibly change their attention and response from one class of neutral stimuli to others (i.e., shifting) was not affected in comparison with the control group. On the contrary, their response was slower when moving their attention from one emotional category (positive or negative word) to the other. The same result has been found in patients with MDD in remission using emotional images (Lange et al., 2012), which suggests that deficits in the ability to flexibly change the focus of attention toward emotional materials may persist as a stable characteristic of depression once the acute phase is over.

In addition to the Go/No-Go Affective Shifting Task, shifting has also been measured using other experimental tasks. Among the first developments on this specific purpose, the shifting task (ST) conceived by Garavan (1998) stands out. Participants are presented with a sequence of stimuli, triangles and rectangles, and are instructed to keep a mental count of both kinds of objects separately. Once each stimulus has appeared, participants are required to update the appropriate mental count for that stimulus and then press a key to move on to the next trial. Garavan's study shows that participants took longer to update one category (e.g., triangles) when the preceding trial required updating the other one (e.g., rectangles) (switch trial) than when the previous trial belonged to the same category (e.g., triangles) (no switch trial). These differences in reaction times were interpreted as an index of the time required to switch attention from internal counting in one category to another, suggesting that the attentional focus capacity is limited (Gehring et al., 2003).

**Figure 1:**
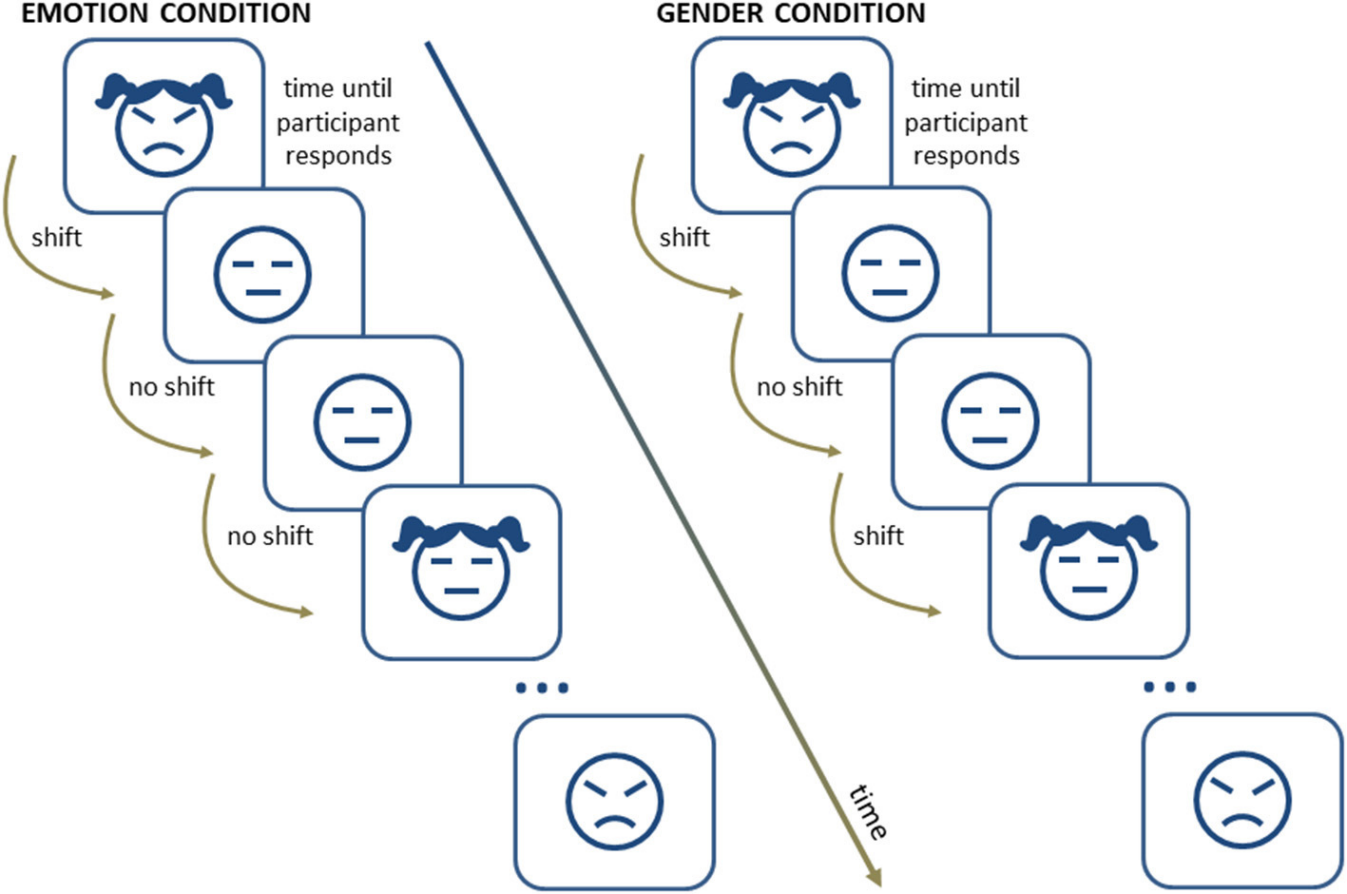
A schematic representation of a series of trials in an internal shifting task.

The ST is also adapted to include emotional stimuli: either words (Chambers et al., 2008; Lo et al., 2012) or facial expressions (De Lissnyder et al., 2012a). The latter is used to study shifting skills in depressed patients (Demeyer et al., 2012) as well as in individuals with depressive symptomatology (Wante et al., 2017). In this internal shifting task (IST), participants perform a mental count of a series of elements, for example, faces, based on their emotional content (negative or neutral) or on the gender of the face (male or female). The so-called “shift cost” (i.e., the difference in reaction times between “shift trials” and “no-shift trials” for both the emotion and the gender conditions) is proposed as a measure of shifting alterations (De Lissnyder et al., 2012a,b) (See Figure 1).

De Lissnyder et al. (2012b) applied the IST paradigm to a group of patients with depression and showed that, compared with a group of healthy volunteers, the patients group was characterized by an overall alteration in their shifting ability (i.e., for both emotional and non-emotional material) rather than by differences related to the emotional condition. Although this study suggests that depression does not particularly affect the ability to shift from one to another emotional information, other studies find a different pattern of results. A study from the same group found specific shifting costs in the emotional condition (Demeyer et al., 2012) in a group of depressed patients in remission. More interestingly, patients with poorer emotional shifting scores during the remission phase showed less clinical improvement after one year compared with those who exhibited higher scores. This result suggests a relationship between the alteration of the shifting component of cognitive control, at least when assessed with emotional stimuli, and the clinical evolution of patients with depression. If confirmed, this type of difficulty might contribute to explain the increased vulnerability to depression after suffering recurrent episodes (De Raedt and Koster, 2010).

In sum, all the reviewed studies report a general alteration in shifting in people with depression. On the contrary, experiments that include emotional content have led to mixed results, some of them suggesting that emotional content acts as an additional burden to the shifting capacity of depressed patients and some others ruling out this possibility. The specific task used to measure this component and certain clinical features, such as the stage of progression of the disorder (i.e., staging) (Guidi et al., 2018) are, therefore, variables to be considered when assessing this effect.

### Updating and Depression

Updating is defined as the ability to exclusively manage and code information that is relevant to the current task or objective among all available information, which requires a continuous replacing of no longer relevant information (Miyake et al., 2000). Thus, updating is deeply related to the monitoring and manipulation of working memory content (Miyake and Friedman, 2012).

The ability to update relevant information in working memory is a critical component of cognition and emotion regulation. People constantly process large amounts of information. To do it efficiently and enable problem solving, goal achievement, and even mood regulation, the content in working memory needs to be fluidly and efficiently updated, maintaining only relevant information (Levens and Gotlib, 2010). Hence, this ability allows individuals to control negative perseverative thinking, removing no longer relevant negative content from working memory (Joormann and Tanovic, 2015). In this sense, difficulties in handling and updating material of negative valence in working memory are associated with both rumination and depression (Joormann et al., 2011).

A review by Snyder (2013) focuses on executive function alterations in patients with depression and concludes that the *n*-back task is the most reported task in the assessment of information updating skills, being used in 7 of 10 studies that address this component. In the classical *n*-back paradigm, participants are presented with a series of letters and are required to indicate whether the current letter is identical or not to the one presented one to three trials back, depending on the specified load. Thus, participants need to maintain and permanently update the relevant pieces of information in working memory. All studies reviewed by Snyder (2013), either using *n*-back tasks or not, confirm the presence of updating deficits in patients with depression compared with healthy controls. Harvey et al. (2004), for example, compares patients with MDD and healthy volunteers in an *n*-back task with three levels of difficulty (i.e., one-, two- and three-back). Contrary to healthy volunteers, patients diagnosed with depression showed worse performance at all difficulty levels. However, patients performed well in the zero-back condition (considered a control measure of attentional processing), which suggests a specific difficulty in the ability to manipulate and update information.

The *n*-back tasks have also been modified to include emotional materials (i.e., emotional *n*-back task). Using faces as emotional stimuli (neutral, happy, and sad), Levens and Gotlib (2010) asked participants to decide whether the face presented showed the same emotional expression as the one presented two stimuli earlier (two-back). The results show that, compared with healthy controls, patients with depression took longer to update working memory representations when these were negative in content (sad facial expression) but were faster in doing so from positive stimuli (happy facial expression). In contrast, healthy participants needed more time to update positive stimuli compared with neutral and negative contents. These differences between groups in reaction times seem to reflect biases in the updating of emotional information in working memory so that healthy volunteers tended to keep positive information in working memory (protective bias) while patients with MDD were more likely to keep negative information (maladaptive bias). It seems plausible to hypothesize, therefore, that biases in updating emotional information may contribute to difficulties in the ability to effectively regulate mood (Levens and Gotlib, 2010). Indeed, indirect evidence from other studies shows that the ability to retain and update positive information in working memory is associated with higher life satisfaction and psychological well-being in healthy individuals (Pe et al., 2013).

Additionally, a modified Sternberg task (Sternberg, 1966) has been commonly used to measure updating skills. In Oberauer's version of the original task, participants are required to memorize two lists of words simultaneously presented. Next, a cue indicates which one of the lists is the relevant one for recognition so that participants are required to update contents in working memory to avoid intrusions from the no longer relevant list (Oberauer, 2001). Joormann and Gotlib (2008) used emotional words in a version of these tasks to estimate working memory updating skills. The authors find that patients with depression experience difficulties updating emotional information in working memory, particularly when that information has negative content. Furthermore, a subsequent study reveals that this deficit seems to be specific to patients with MDD and is not generalizable to other types of disorders such as social anxiety disorder (see also Yoon et al., 2014).

In conclusion, it seems that patients with MDD present an altered performance in their ability to update relevant information in working memory, particularly when the information that is no longer relevant has emotional content of negative valence.

### Inhibition and Depression

Cognitive inhibition is a complex executive control mechanism that comprises different elements (Friedman and Miyake, 2004). It can be described as the process of preventing the activation of irrelevant or unnecessary information as well as the ability to restrict and ignore such activation by updating the content of working memory (Harfmann, 2016). Inhibition also includes the ability to suppress or avoid automatic and dominant responses to activate less automatic but relevant responses to our goals (Miyake et al., 2000). Ignoring and inhibiting the processing of irrelevant information that captures our attention is a fundamental ability that allows us to generate flexible responses and adjust our behavior and our emotional responses to the current situation (Joormann, 2010). Indeed, it has been proposed that people with depression face difficulties when ignoring and removing irrelevant negative material from working memory (Joormann, 2010).

Inhibitory deficits in patients with MDD have been explored by classical neuropsychological tests such as the Stroop test (Stroop, 1992). The “Stroop interference effect” refers to longer response times when the name of a word and the color in which the word is written are different (e.g., “red” written in blue) compared with when they are congruent. In administering this task to a population with depression, slower overall performance is found in comparison with healthy controls. Patients also present greater interference effects (Stordal et al., 2005; Markela-Lerenc et al., 2006; Gohier et al., 2009). Furthermore, a longitudinal study using the Stroop test reveals that impairments in inhibitory processes are maintained over time (up to 10 years) in individuals with recurrent depression and that those impairments are predictive of poor functioning (Årdal and Hammar, 2011). Thus, impaired inhibitory processes might be considered a potential vulnerability marker for depression.

The Stroop test has also been modified to include emotional material. In the “emotional Stroop test,” words in different colors have a positive, negative, or neutral affective valence. As in the original version, participants must name the color in which the word is written regardless of its emotional valence. Performance in this modified task is associated with worse performance on non-valenced versions of the task but only for those individuals within a depressive episode at the moment of the assessment (Hsu and Davison, 2017). Using this task, Dai and Feng (2011) report greater interference effects in words with negative valence in patients with MDD compared with healthy participants. More interestingly, this difference is also found when patients with MDD were compared with a group of patients with MDD in remission in accordance with previous evidence from the Negative Affective Priming task (see below) (Joormann and Gotlib, 2010). Finally, a meta-analysis on the effects of the emotional Stroop task in patients with MDD confirms these results and reveals that the emotional Stroop effect is modulated by the degree of severity of depression so that individuals with more severe symptoms show longer response latencies to stimuli with negative and positive valence as compared with latencies to neutral stimuli (Epp et al., 2012). Furthermore, recent evidence suggests that depressed individuals show a relative attentional disengagement from positive compared with negative stimuli in the emotional Stroop task (Loeffler et al., 2019).

To assess the ability to inhibit irrelevant information, different experimental tasks, mainly based on the phenomenon of “negative priming,” are also used. The term “negative priming” describes the inhibitory effect of an ignored stimulus in contrast to the facilitating effect (priming) generated by the attended stimulus (Neill et al., 1995). The “negative priming paradigm” allows estimating the power of inhibitory processes based on the delayed response latency to a target when the distractor from a previous trial becomes the target in the present trial (Joormann, 2006). Different population groups, such as older adults, children, or patients with schizophrenia, show deficits in negative priming, which illustrates difficulties in inhibiting the intrusion of irrelevant information in working memory (Joormann, 2006).

**Figure 2:**
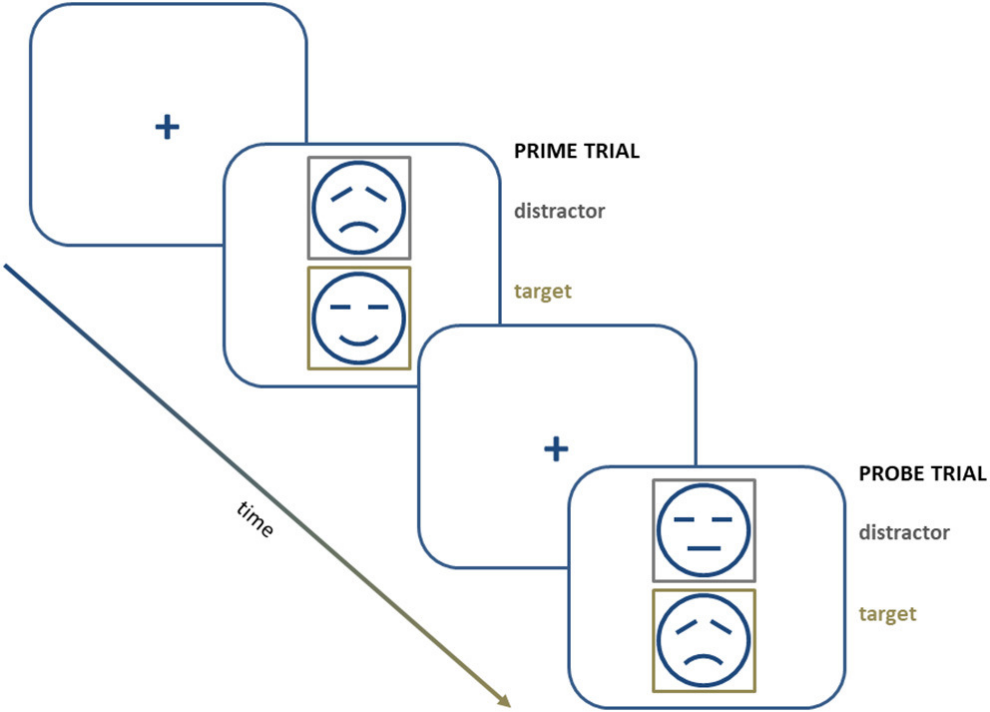
A schematic representation of a series of trials in a negative affective priming procedure.

This paradigm is also adapted to be used with emotional material (“negative affective priming”; NAP). This modification also comprises “prime” and “probe” trials, in which two emotional stimuli (e.g., faces or words) are presented simultaneously as targets or distractors, according to the color of their frame. Participants are instructed to evaluate the valence of the target stimulus and ignore the distractor stimulus (Figure 2). Increased reaction times when the distractor stimulus shares its valence with the target stimulus are taken as an index of a negative priming effect. In other words, such an increase reflects the inhibition of the affective material during the prime trial (Harfmann, 2016).

Goeleven et al. (2006) use this design to explore the ability to inhibit sad or happy facial expressions in depression. Patients with MDD show a reduced or even reversed “NAP effect” compared with healthy controls in tests with emotional material of negative valence. However, the ability to inhibit emotional material of positive valence is not affected. This result suggests that, at least with this type of procedure, depressed patients have difficulties specifically inhibiting negative information.

Frings et al. (2007) replicated the study by Goeleven et al. (2006) in dysphoric individuals (i.e., healthy individuals reporting mild levels of severity of depression symptoms). Their results show that only participants with no depressive symptoms exhibited the NAP effect for negative emotional material. On the contrary, dysphoric participants showed a reversed NAP effect for negative emotional material and a significant NAP effect for positive material. These results suggest that inhibitory deficits previously reported in patients with MDD can also be observed in individuals free of pathology who experience depressive symptoms (Frings et al., 2007). In this line, a study by Joormann and Gotlib (2010) found that, whereas patients with a current diagnosis of MDD experienced deficits in inhibiting negative emotional information, patients with MDD in remission showed a significant NAP effect toward non-relevant material of negative valence. These results suggest that deficits in the inhibition of negative information could be only present in active stages of the disorder.

Complementary evidence on the role of inhibitory deficits in depression comes from active forgetting experiments. Although remembering positive events and forgetting negative ones has been associated with adaptive repairing strategies from negative mood (Joormann and Siemer, 2004) and with increased well-being across the life span (Charles et al., 2003), meta-analytic evidence shows that patients with clinical depression preferentially recall negative over positive materials presented in experimental tasks (e.g., Matt et al., 1992). Several authors propose that this phenomenon might be sustained by inhibitory deficits that prevent these patients from suppressing negative memories. Suppressing unwanted memories can be attempted either during memory encoding or retrieval (see Anderson and Hanslmayr, 2014 and Anderson and Hulbert, 2021, for a review). Evidence from directed forgetting studies, in which participants are required to inhibit memories that have just been acquired (see Bjork, 1989), suggests that depressed patients experience difficulties at suppressing to-be-forgotten items in these tasks (Cottencin et al., 2008). More interesting, directed forgetting deficits seem to be evident for negative items, and directed forgetting of positive or neutral items remains unaffected (Power et al., 2000; Berman et al., 2011; Yang et al., 2016; Xie et al., 2018). These difficulties are not limited to the initial process of learning and storing materials. Forgetting difficulties in depressed patients also have been reported for memories already consolidated. Most people are able to control and limit the entrance of stored but unwanted memories into awareness by stopping, whether automatically or voluntarily, their retrieval. Indeed, this ability is proposed as a fundamental ingredient for adaptive emotion regulation because avoiding access to such memories into awareness also limits the affective reactions associated with the memories (see Engen and Anderson, 2018). Nevertheless, studies using the think/no-think paradigm have led to inconsistent findings. Although Joormann et al. (2009) report reduced forgetting of negative words, a finding that has been replicated for pictorial stimuli in individuals with depressive symptomatology (Zhang et al., 2016), other studies fail to replicate these findings (Joormann et al., 2005; Sacchet et al., 2017). Interestingly though, forgetting of unwanted memories in those studies were accompanied by increased suppression-related brain activity (Sacchet et al., 2017) or by a general deficient recall (Joormann et al., 2005), suggesting that depressed individuals might achieve forgetting of unwanted negative memories at higher neurocognitive cost.

Thus, the extant evidence supports the idea that patients with MDD also present deficits in the inhibition component of cognitive control. These deficits are more evident when the inhibited materials have negative valence, which is in line with what has been observed for the other cognitive control components.

Overall, studies so far reveal cognitive control alterations in depression as manifested by difficulties in shifting from one task to another, in updating necessary and relevant information for a given goal, and in inhibiting irrelevant information for such purpose (e.g., negative information in the case of emotion-regulation processes). Separate analyses of these cognitive control components suggest that, in general, patients with MDD show deficits in all of them, mainly in relation to longer reaction times. Specifically, a recent study analyzing the three cognitive control components suggests that patients with MMD present greater difficulty shifting away from an emotion-relevant task set (compared with an emotion-irrelevant one), updating WM with emotional contents, and a reduced ability to inhibit the processing of negative distracting stimuli (Quigley et al., 2020). Whether these deficits are modulated by the severity of the condition or whether they remain or disappear at remission is still uncertain given the inconsistency of the findings available so far.

## Cognitive Control Difficulties and Severity of Depression

Research on cognition and depression has explored whether cognitive deficits are significantly associated with clinical variables. As previously mentioned, some studies find that factors such as severity of the disorder may modulate performance in tasks aimed at measuring cognitive control (e.g., Everaert et al., 2017b).

However, a closer examination of the concept of “severity” of depression reveals a lack of consensus on its definition, which could affect the interpretation of the results. A previous literature review (McClintock et al., 2010) shows that the heterogeneity in the degree of cognitive impairment observed in patients may depend, in part, on the index of severity used in the studies (e.g., number and duration of depressive episodes, duration of the disorders, resistance to treatment, or presence of other comorbid disorders). For example, patients with recurrent depressive episodes suffer greater cognitive impairments in attention, memory, processing speed, and executive function than first-episode patients (Karabekiroglu et al., 2010; Gonda et al., 2015; Talarowska et al., 2015; Semkovska et al., 2019) although other studies have not been able to replicate these effects (Lee et al., 2012; Roca et al., 2015, 2016).

One of the most common ways to characterize depression severity is based on total scores in standard self-reported symptoms. A review by Pantzar et al. (2014) suggests that patients with severe or moderate depression exhibit worse performance in different tasks related to processing speed, attention, executive function, verbal fluency, episodic memory, and vocabulary compared with mildly depressed patients, who only show alterations in speed of processing. However, the evidence shows mixed results as subsequent studies have not revealed an effect of depression severity on the cognitive performance of adult patients of MDD (Albert et al., 2018; see also MacKin et al., 2014).

Regarding cognitive control specifically, Everaert et al. (2017b) show that depression severity is related to greater alterations in global cognitive control. Studies addressing the relation between the severity of depressive symptomatology and impairments in the different components of cognitive control are scarce and show certain heterogeneity. However, on the whole, there seems to be a positive relationship between deficits in the cognitive control components and the severity of depressive symptomatology.

A review by Snyder (2013), analyzing the studies that until then had used the WCST and the TMT-B measures, shows that performance worsened as the severity of depression increased but only in the WCST and not in the TMT-B. This lack of correlation between severity of depression and performance in the TMT-B could be due to an overall slowing effect in processing speed, present in part A of the same test, which could have masked specific shifting effects.

Similarly, there is also some variability in the influence of MDD symptom severity on the ability to update information in working memory. Using an *n*-back task, Harvey et al. (2004) found that subjects with MDD had problems updating information in working memory. Although symptom severity was not associated with alterations in this cognitive control component, a higher number of hospital admissions was found to be related to poorer updating skills (Harvey et al., 2004). These conflicting results suggest that using measures that are sensitive to changes or group differences (Guidi et al., 2018) is a more reliable strategy to study the linkage between severity of depression and cognitive deficits and biases (McClintock et al., 2010).

Unlike literature on the relation between severity of depression and global cognitive control difficulties, specific evidence on the association between depression severity and inhibition difficulties seems robust. In a systematic review, Epp et al. (2012) concludes that more severe symptomatology (comparing clinically depressed patients, dysphoric patients, people with an induced negative mood and controls) was associated with worse performance in the emotional Stroop task. Likewise, the slowing in incongruent trials with words of negative valence is related to the disorder severity (Stolicyn et al., 2016). This association is also found in studies analyzing difficulties in emotion regulation in depression. For instance, Kahn et al. (2019) find that emotion reactivity to happy or sad film clips shows a non-linear association related to the severity of depression symptoms. More specifically, from low levels of depressive symptoms to approximately the balanced clinical cutoff, the growth in sadness-expressive experience increased with depression symptoms. Yet, at higher levels of depression, emotional reactivity is reduced.

In sum, studies that address the influence of the severity of depression on cognitive control deficits in patients with depression are scarce, and their outcomes reflect significant heterogeneity. Part of the variability observed in this set of studies could be attributed to the way in which the different authors have measured the severity of the disorder, from scales and standardized questionnaires (Epp et al., 2012; Snyder, 2013) to the use of other sorts of clinical information, such as number of hospital admissions (Harvey et al., 2004). Also, the standard methodological procedure of using a total score in a scale to assess overall severity is inadequate to detect possible subtle connections between cognitive control components and specific symptoms or groups of symptoms (Fried and Nesse, 2015). Nonetheless, taken together, these studies suggest that the degree of impairment in the different cognitive control components is partially associated with the severity of the disorder, so individuals with lower cognitive control capacities tend to experience more severe symptoms of depression.

## Rumination and Depression

The present review focuses on reviewing the evidence that may connect cognitive biases and cognitive control difficulties in depression. On one hand, cognitive biases are basically related to specific ways of processing, storing, retrieving, and interpreting negative or positive information (Gotlib and Joormann, 2010). On the other hand, cognitive control is related to the ability to achieve complex goals efficiently and flexibly. A third cognitive component has to do with modes of processing (i.e., styles of dealing with information independent of the contents being processed). Perhaps the most extensively researched mode of processing in depression is rumination (Nolen-Hoeksema, 1991). Depressive ruminations, according to the seminal response styles theory proposed by Nolen-Hoeksema (2004), are defined as the way to respond to feelings of distress by repetitively and passively focusing on the negative symptoms and their possible causes and consequences (Nolen-Hoeksema et al., 2008). Although it can be considered an emotion-regulation strategy (Aldao et al., 2010), it also can be conceptualized as a specific tendency to process information (De Raedt et al., 2015). In fact, many experimental studies show that rumination can be experimentally manipulated under controlled conditions. For instance, it is shown that rumination inductions worsen negative mood in clinically depressed participants (Donaldson and Lam, 2004) and slow down recovery from an induced sad mood in healthy individuals (Zetsche et al., 2009). It is found that a tendency to engage in a repetitive or ruminative mode when handling negative cognitive contents is associated with deficits in cognitive control (Joormann and Quinn, 2014; Joormann and Vanderlind, 2014; Joormann and Stanton, 2016). Although the exact relation between rumination and impaired cognitive control is still relatively unknown, several authors suggest that there are several possible pathways by which those elements could be interconnected. Koster et al. (2011) propose that an impaired ability to exert attentional control toward negative stimuli or events may interfere with the ability to disengage from them and, subsequently, may worsen mood. Also, these cognitive control deficits may help to perpetuate negative cognitive habits (i.e., biases in attention, interpretation, and memory for emotional information; see Hertel, 2004).

**Figure 3:**
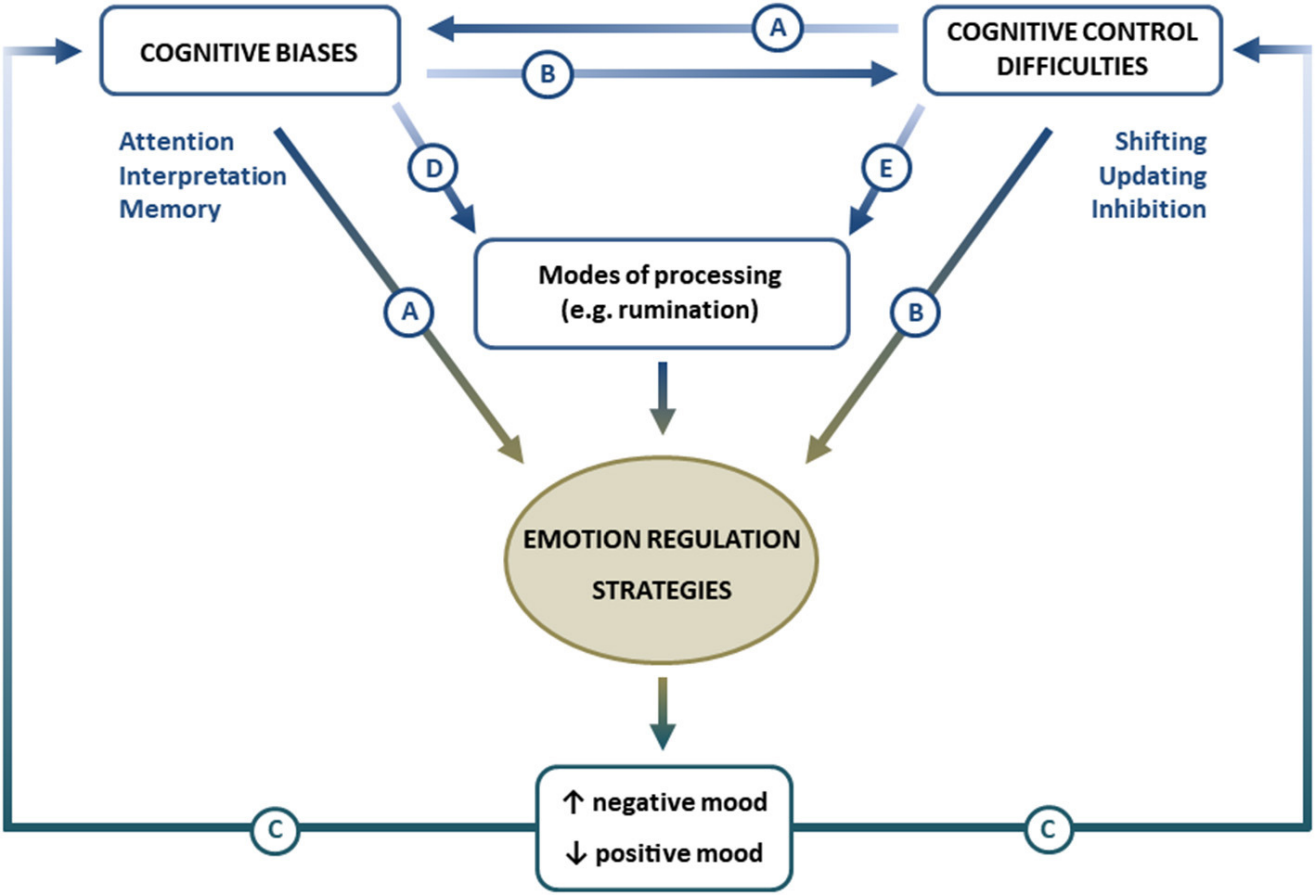
Proposal for an integrative interplay model among cognitive control, cognitive biases, and emotion regulation in depression.

Whereas repetitive thinking may not necessarily have a negative impact on mood, research has found that, when that style of processing is coupled with negative cognitive biases (e.g., selective sad memories or negative interpretations) about oneself or the world, that combination predicts increases in negative daily mood (Poerio et al., 2013) and the onset of clinical episodes of depression (Alloy et al., 2006) as well as other psychopathological conditions (Joormann and Tanovic, 2015). Thus, it is plausible that deficits in cognitive control may make it difficult for depressed individuals to disengage from repetitive negative thoughts (Hertel, 2004), which, in turn, may diminish the use of adaptive emotion-regulation strategies such as reappraisal (Figure 3).

As previously mentioned, depressive symptomatology is associated with the impairment of different cognitive processes, among which processing speed, attention, memory, and executive function stand out (Marazziti et al., 2010). Moreover, patients with depression and a tendency to ruminate experience greater deficits in processing speed and executive function than their counterparts with low rumination tendency (Schwert et al., 2017).

Numerous studies on depression reveal significant associations between the tendency to ruminate and an altered performance in different components of cognitive control. In particular, low performance in IST tasks seems to be associated with the presence of ruminations (De Lissnyder et al., 2012a,b). In other words, patients with MDD who experience repetitive negative thoughts or images also present concomitant difficulties in tasks that require control over mental representations held in working memory. Moreover, these relationships seem to occur as well in people with depressive symptomatology, but without an MDD diagnosis, particularly when such representations have a negative emotional valence (De Lissnyder et al., 2012a). Given that the presence of ruminations is considered a risk factor for the development of depression (Joormann and Tanovic, 2015), it is plausible that cognitive control deficits associated with rumination could be already present before the development of the disorder.

Similarly, the ability to update information in working memory has also been related to the presence of rumination in patients with MDD (Joormann et al., 2011). As previously mentioned, these patients experience difficulties in removing negative non-relevant information from working memory. In addition, depression is associated with a greater tendency to dwell on ruminations (Joormann and Gotlib, 2008). In particular, higher levels of “brooding” rumination (a particularly maladaptive modality of rumination characterized by perseverative, passive, and judgmental focus on one's mood) are associated with the greatest difficulties in the updating component (Yoon et al., 2014). Indeed, a recent metanalysis by Zetsche et al. (2018) covering anxiety and depression conditions finds that the only cognitive control component associated with rumination (or worry) is difficulties in discarding irrelevant material from working memory although the overall magnitude of the effect was modest (*r* = −0.20).

Finally, inhibitory deficits in patients with MDD, in particular, the ability to inhibit emotional material of negative valence, seem also to be related to a tendency to ruminate (Joormann and Gotlib, 2010). Inhibition can operate in different phases during information processing by limiting the entry of information to working memory (i.e., “interference control”) or by removing information that has already accessed working memory but is no longer relevant. Patients with MDD show difficulties controlling the entrance of negative information into working memory (Zetsche and Joormann, 2011). However, the tendency to experience ruminative thoughts is more related to difficulties in suppressing negative information that has already accessed working memory. As previously commented, experiencing repetitive thoughts is a normal response to stressful or unpleasant events. It is the inability to naturally disengage from negative thoughts that makes it a maladaptive strategy (Koster et al., 2011). Therefore, it appears that cognitive deficits in patients with a rumination tendency mainly affect their ability to suppress these thoughts rather than their ability to control the initial access to working memory (Zetsche et al., 2012). Intrusive thoughts may also come from long-term memory in the form of unwanted memories. Healthy individuals are usually able to short circuit the retrieval of intrusive memories, leading to a suppression-induced forgetting of those unwanted memories (see Anderson and Huddleston, 2012 and Anderson and Hulbert, 2021 for reviews). On the contrary, individuals scoring high in rumination experience difficulties to inhibit intrusive thoughts in suppression induced forgetting paradigms (Fawcett et al., 2015).

Overall, the evidence reviewed suggests a relationship between alterations in different components of cognitive control (shifting, updating, and inhibition) and a tendency to process information in a repetitive way although there is some controversy on the cognitive control components most closely related to rumination (Yang et al., 2017; Zetsche et al., 2018). Cognitive difficulties in depression may result in a sort of cognitive bottleneck (e.g., difficulty getting rid of negative or intrusive materials) that hinders the efficiency of information processing. Rumination can also be triggered or exacerbated by emotional experiences and difficulties in regulating them. In a series of studies, Hervas and Vazquez (2011) showed that sad mood is often accompanied by other negative emotions that are simultaneously experienced or raised along with that mood. Interestingly, this “emotional overproduction” state mediates the relationship between neuroticism and rumination. In other words, it is possible that the tendency to ruminate is due not only to cognitive control or personality factors (such as neuroticism), but to difficulties adequately regulating emotions once they are initiated. An adequate functioning of the different components of cognitive control seems to be crucial to maintaining emotion regulation skills, limiting and controlling the effect of negative mood and associated negative emotions. Thus, deficits in cognitive control, in association with a ruminative mode of processing, appear to contribute to the onset and maintenance of depression through emotion-regulation difficulties (Von Hippel et al., 2008).

## Conclusions: Toward an Integrative Interplay Model

To date, numerous studies have explored each cognitive control component (i.e., shifting, updating, and inhibition) as proposed by Miyake et al. (2000) in a variety of samples (dysphoria, mood induction, or clinical depression), either through experimental tasks or using classical neuropsychological tests. Our review reveals that most of these studies confirm the existence of cognitive control difficulties in these patients, particularly when the material has emotional valence. Although further research is needed to disentangle whether these difficulties are a state marker of depression that would disappear after recovery from the depressive episode (Joormann and Gotlib, 2010) or otherwise persist in remission (Årdal and Hammar, 2011; Gupta and Kar, 2012; Semkovska et al., 2019), the extant evidence reviewed here points to this higher cognitive function playing a fundamental role in the initiation and maintenance of depressive symptomatology (Grahek et al., 2018; LeMoult and Gotlib, 2019).

Overall, it seems that depressed individuals show alterations in components of cognitive control, and more interestingly, these difficulties seem to be more distinctive when cognitive control is exerted over negative emotional material. This mood-congruent effect is consistent with the well-established evidence on processing biases in depression (Mathews and MacLeod, 2005; Teachman et al., 2012), which suggests the existence of a “functional” relationship between cognitive deficits and cognitive biases. The mutual relationship between cognitive deficits and biases might be a fundamental basis for the onset and maintenance of depressive symptoms (Everaert et al., 2017b; Grahek et al., 2018). Still, despite some authors have claimed the need to integrate the literature on general cognitive deficits and biases in depression (Gotlib and Joormann, 2010), studies exploring the nature and the causal relationships of these interactions are scarce (Everaert et al., 2017b).

In light of the reviewed literature, two alternative proposals can be made. First, it could be argued that depressed people are characterized by a general deficit in cognitive control that modulates cognitive biases, thus causing and maintaining depressive symptoms – i.e., cognitive control difficulties → cognitive biases → emotion-regulation difficulties → symptoms of depression; see Figure 3, path A. This pathway proposes that a primary cognitive control deficit would contribute to the development and maintenance of cognitive biases that, in turn, via emotion-regulation strategies, modulate depressive symptoms. This hypothesis has received recent support in a study showing an indirect effect of impairment in shifting, updating, and inhibition on depressive symptoms through attention and/or interpretation biases (Everaert et al., 2017b). The initial element of this hypothesized pathway is an overall cognitive control deficit in depressed people, irrespective of the valence of the task. However, these initial difficulties in shifting, updating, and inhibiting neutral information (Harvey et al., 2004; Stordal et al., 2005; Markela-Lerenc et al., 2006; Gohier et al., 2009; Snyder, 2013; Lin et al., 2014; MacKin et al., 2014) might be particularly evident when executive control is exerted over negative stimuli (see, for example, Goeleven et al., 2006; Joormann and Gotlib, 2008, 2010; Joormann et al., 2009; Levens and Gotlib, 2010; Berman et al., 2011; Dai and Feng, 2011; Demeyer et al., 2012; Epp et al., 2012; Murphy et al., 2012; Wante et al., 2017), which, in turn, might feed or maintain cognitive biases toward negative emotional information. To complete this pathway, it could be possible that cognitive biases also have a direct influence on emotion regulation. For instance, an eye-tracking study in which a depressed mood was induced in healthy volunteers shows that the more time participants looked at happy faces (presented in pairs with neutral or sad faces), the faster the recovery from the negative mood was (Sanchez et al., 2014).

Alternatively, it may be argued that the presence of processing biases in depression can act as a burden for cognitive control operations. This pathway (see Figure 3, path B) suggests that cognitive biases → cognitive control difficulties → emotion-regulation difficulties → symptoms of depression. Paying more attention to negative information or a tendency to remember more negative than positive information, for instance, might reduce the capacity to shift from negative information to positive or non-emotional information, to update negative contents in working memory, and to inhibit the processing of negative stimuli while cognitive control of non-emotional material should be relatively preserved. Some previous models of depression already hypothesized that paying attention to affective information may reduce the capacity to effectively process other types of information simultaneously presented (Hertel, 2004). Complementing this affective interference hypothesis (see Gotlib and Joormann, 2010), here we review extensive evidence in favor of deficits in shifting, updating, and inhibition, that are most evident when the material to be controlled is negative. Furthermore, some studies report impairments in cognitive control of positive material that are consistent with the “positive blockade” proposed by Disner et al. (2011) (see also Levens and Gotlib, 2010).

However, paths A and B can coexist. According to those studies that show general cognitive control deficits affecting non-emotional information and also considering that cognitive control impairments have been more frequently reported for mood-congruent material, it could be the case that people suffering depression have a primary, perhaps subtle, deficit in their cognitive control abilities, which could be boosted by cognitive biases. The interplay of both features would, thus, be a fundamental mechanism for the onset and maintenance depressive mood.

Our proposed model also reflects the possibility that mood may fuel both cognitive biases and cognitive control difficulties—see Figure 3, path C. Depression is characterized by a negatively biased interpretation of ambiguous information (Everaert et al., 2017b), by a difficulty in disconnecting from negative material that has captured attention or entered working memory, and by difficulty in the ability to use positive autobiographical memories to modify negative mood (LeMoult and Gotlib, 2019). Experiencing negative mood is usually associated with the activation of representations in working memory that are congruent with such mood (Siemer, 2005). Thus, negative mood is related to a more frequent presence of negative thoughts, selective attention to negative stimuli, and greater access to negative memories (Joormann and Gotlib, 2010). It is in this context that the capacity for cognitive change, working memory updating, and inhibition of irrelevant information proves to be fundamental as cognitive control abilities are key components for adaptive emotion regulation (Joormann, 2010). Moreover, a balanced functioning of all different components of cognitive control could be relevant for adaptive emotion regulation as the flexible use of different emotion-regulation strategies (Stange et al., 2017) is crucial to adapt to changing conditions of the environment (Bonanno and Burton, 2013; Aldao et al., 2015; Joormann and Stanton, 2016). Consequently, minor difficulties in cognitive control, otherwise unnoticed, may become evident when individuals have to deal with bias-congruent information. These mutual influences (see Figure 3) may contribute to sustain negative affect and, what has been relatively neglected in previous models of cognition and depression, to deficits associated with generating or sustaining positive affect (Joormann and Vanderlind, 2014).

Our integrative model considers that modes of processing, in particular, a tendency to ruminate, are also relevant and have significant associations with attentional biases (Whitmer and Gotlib, 2013) or autobiographical memory biases (Romero et al., 2014; Sumner et al., 2014)—see Figure 3, path D. Rumination is repeatedly found to be associated with the onset and recurrence of depressive episodes (Nolen-Hoeksema et al., 2008), and more importantly, recent research finds that it is significantly associated with cognitive control deficits—see Figure 3, path E. The reviewed literature shows that a repetitive style of thinking is related to difficulties in shifting (De Lissnyder et al., 2012a,b; Vergara-Lopez et al., 2016), updating (Joormann and Gotlib, 2008; Yoon et al., 2014), and inhibition (Joormann and Gotlib, 2010; Zetsche and Joormann, 2011; Zetsche et al., 2012) in individuals with depression (see also Zetsche et al., 2018; Sanchez-Lopez et al., 2019; Yaroslavsky et al., 2019). Therefore, it seems plausible to argue that, in patients presenting difficulties disengaging their attention from emotional stimuli, to change the course of thought, control the working memory content, or remove irrelevant unpleasant thoughts, a repetitive mode of processing may make it difficult to adequately regulate their emotion responses, thereby contributing to intensifying the negative mood (Nolen-Hoeksema, 1991) and influencing symptom severity and episode recurrence (Nolen-Hoeksema et al., 2008).

Severity is an important clinical factor that has been explored regarding its connection to deficits in cognitive control and depression. Despite the heterogeneity of the severity measures used by different authors (McClintock et al., 2010) and the lack of empirical work regarding alterations in cognitive control components with the severity of symptoms, the reviewed evidence suggests an association between impairments in shifting (Snyder, 2013), updating (Harvey et al., 2004), and inhibition (Epp et al., 2012; Stolicyn et al., 2016) and greater disorder severity. Further studies should explore whether specific symptoms or a constellation of symptoms beyond overall severity are particularly associated with each component of the model.

Most of the studies reviewed here provide evidence supporting a critical role of cognitive control deficits in depression. Furthermore, and more relevant to our proposed model, many of them suggest significant interactions between cognitive control impairments, cognitive biases, and rumination for the development and maintenance of depressive symptoms. However, there are still important issues that limit our ability to draw conclusions that need to be carefully considered. For this review, the three-component model of cognitive control proposed by Miyake and colleagues (Miyake et al., 2000) was chosen as a framework around which the review of the extant knowledge could be articulated. Even though this conception of cognitive control has inspired many empirical studies in experimental psychology and in the clinical field (Quigley et al., 2020), the model itself is not exempt from limitations. As previously commented, recent studies propose modifications to the original structure derived from confirmatory factor analysis (see Miyake and Friedman, 2012; Friedman and Miyake, 2017 for a detailed discussion). Besides this, it is proposed that the original structure may not be completely adequate when cognitive control needs to be exerted over emotional material (Everaert et al., 2017b; Grahek et al., 2018).

Another important consideration should be made in terms of sensitivity, reliability, and purity of neuropsychological and experimental tasks for the assessment of cognitive control. Most of the neuropsychological tasks in the studies reviewed here were developed to detect mild-to-severe neuropsychological impairments and might not be sensitive enough to detect subtle changes in psychopathological conditions, such as depression. In addition, the reliability of measures is an issue hardly addressed in most studies on cognitive control in depression even though there is some evidence suggesting that the reliability of most measures is limited (Hedge et al., 2018; Zetsche et al., 2018). Finally, our capacity to measure components of cognitive control precisely is affected by the presence of non-cognitive control processes associated with the task (see Snyder et al., 2015 for more detailed discussion).

Also important from a methodological point of view, the vast majority of studies on cognitive deficits and depression use cross-sectional methodologies or, in some cases, longitudinal studies measuring performance on few occasions (e.g., before and after treatment). Studies exploring the dynamics of cognitive deficits and symptoms in depression are needed. For instance, using experience sample methodologies (i.e., diaries or momentary assessments), it is found that the activation of a negative emotion (e.g., sadness) in depressed individuals easily activates other negative emotions. This propagation effect or “inertia” (Pe et al., 2014; Hayes et al., 2015) seems to be a core element of sustained negative effects in depression (Brose et al., 2015). It could be possible that momentary or permanent activation of specific cognitive factors (e.g., switching difficulties) might play a role in this negative emotional cascade. Although there is no direct evidence that this could be the case in depression, Heeren and McNally (2016) show that the orienting component of attention in combination with symptoms of avoidance and fear of social situations are mutually interconnected and centrally situated in the network of symptoms of individuals with social anxiety disorder. Thus, a new generation of studies using more robust assessment methods, more sophisticated analytic procedures (e.g., network analyses), and more adequate designs to capture mutual dynamic relations between symptoms and cognition (e.g., ecological momentary assessment) (Yaroslavsky et al., 2019) could shed more light on the complex relationships between cognitive capacities, cognitive biases, emotion regulation, and depression.

Finally, other factors need to be considered before drawing conclusions on cognitive control abilities in depression. It is often ignored in neuropsychological testing that patients with depression show a more negative attitude toward testing, lower performance motivation, and more negative momentary influences, all of which can contribute to poor performance that could be mistakenly attributed to actual cognitive control deficits (Moritz et al., 2017). In addition, the potential effect of pharmacological treatments for depression deserves consideration. Current treatments are known to improve mood symptoms, but some studies show that antidepressant treatment itself can negatively affect certain aspects of cognitive performance, such as memory, executive function, or processing speed (McClintock et al., 2010) even in remitted patients who are still under treatment (Nagane et al., 2014). By contrast, other studies suggest that treatment with antidepressants significantly enhances objective and subjective measures of cognitive functions in adults with recurrent MDD, independently of improvements on depressive symptomatology (McIntyre et al., 2014; Jonassen et al., 2015). In any case, the scarcity of evidence on medication effects on specific cognitive functions in MDD makes it difficult to draw conclusions in regard to their potential influence on cognitive control functioning (Kaser et al., 2017).

Derived from the aforementioned limitations of the study regarding the role of cognitive control in depression, an alternative approach to the problem has been recently proposed by Grahek et al. (2018). In their view, deficits in cognitive control in depression would not stem from an attenuated ability to exert cognitive control *per se*. Instead, impairments would arise from a deficient ability to detect when, with what intensity, and for how long to engage in controlled processing of information. In this view, cognitive control could be considered as a systematic failure to effectively engage in controlled processing that supports goal-directed behavior (Grahek et al., 2018). This is an interesting hypothesis related to monitoring cognitive efforts that needs further research.

Despite the difficulties dissecting the role of specific cognitive control impairments in the onset and maintenance of depressive symptoms, the literature reviewed here clearly highlights the role of those deficits in association with cognitive biases and modes of processing. Based on these new insights, the development of integrative intervention approaches that consider the mutual influences of different cognitive components on mood regulation and symptoms of depression seems foreseeable. Cognitive modification of emotion regulation can be achieved through different pathways (see Gotlib and Joormann, 2010). For instance, recent efforts have been devoted to developing effective cognitive control training programs that, in combination with evidence-based interventions, could contribute to breaking down the dysfunctional emotion-regulation strategies by training cognitive control over bias-congruent material (Roiser et al., 2012; Kaser et al., 2017). Recent research in this direction is showing promising results, suggesting that cognitive control training in MDD patients is associated with moderate effects in their symptom severity and daily functioning (Motter et al., 2016; see Dolcos et al., 2019 for a review). If the proposed model (Figure 3) is correct, then future developments in cognitive control training might conveniently assess and intervene not only in the functioning of “cold” cognitions, but also indirectly help to correct “hot” cognitions. Also, cognitive bias modification procedures (i.e., cognitive training aimed at modifying cognitive biases in attention, memory, or interpretation) that have shown limited effects in changing either mood or cognitive biases in depression (Jones and Sharpe, 2017) might enhance their results if they were to address specific cognitive control deficits when designing the training tasks. In this direction, metacognitive therapy (Gupta, 2007; Wells, 2009; Normann and Morina, 2018) that is aimed not to directly change cognitive biases, but to help patients to relate to these biases in a new way (e.g., using mindfulness techniques and attentional control techniques), fits well with the evidence presented in our model. Similarly, cognitive trainings directly aimed at reducing rumination (e.g., Watkins, 2009), may also positively affect cognitive control functioning and/or cognitive biases through the purported interconnections described in our model. In sum, there is a need to optimize integrative interventions by taking advantage of the two different cognitive traditions considered in the present review. That would probably allow effectively reducing symptoms as well as relapses and recurrences in one of the psychological disorders causing most suffering in humans.

## Author Contributions

DV conducted the literature search and provided summaries of previous research studies. DV and JP wrote the first draft of the manuscript and contributed to the next developments. CV strongly contributed to the progress of the manuscript and provided an expert clinical perspective. All authors contributed to and approved the final version of the manuscript.

## Conflict of Interest

The authors declare that the research was conducted in the absence of any commercial or financial relationships that could be construed as a potential conflict of interest.
